# Carrot Anthocyanins Genetics and Genomics: Status and Perspectives to Improve Its Application for the Food Colorant Industry

**DOI:** 10.3390/genes11080906

**Published:** 2020-08-07

**Authors:** Massimo Iorizzo, Julien Curaba, Marti Pottorff, Mario G. Ferruzzi, Philipp Simon, Pablo F. Cavagnaro

**Affiliations:** 1 Plants for Human Health Institute, North Carolina State University, Kannapolis, NC 28081, USA; jbcuraba@ncsu.edu (J.C.); mopottor@ncsu.edu (M.P.); mferruz@ncsu.edu (M.G.F.); 2 Department of Horticultural Science, North Carolina State University, Raleigh, NC 27695, USA; 3 Department of Horticulture, University of Wisconsin–Madison, Madison, WI 53706, USA; philipp.simon@usda.gov; 4 Vegetable Crops Research Unit, US Department of Agriculture–Agricultural Research Service, Madison, WI 53706, USA; 5 National Scientific and Technical Research Council (CONICET), National Agricultural Technology Institute (INTA) E.E.A. La Consulta, Mendoza 5567, Argentina; pablocavagnaro@hotmail.com; 6 Faculty of Agricultural Sciences, National University of Cuyo, Mendoza 5505, Argentina

**Keywords:** anthocyanins, carrots, natural colorant, genetics

## Abstract

Purple or black carrots (*Daucus carota* ssp. *sativus* var. *atrorubens* Alef) are characterized by their dark purple- to black-colored roots, owing their appearance to high anthocyanin concentrations. In recent years, there has been increasing interest in the use of black carrot anthocyanins as natural food dyes. Black carrot roots contain large quantities of mono-acylated anthocyanins, which impart a measure of heat-, light- and pH-stability, enhancing the color-stability of food products over their shelf-life. The genetic pathway controlling anthocyanin biosynthesis appears well conserved among land plants; however, different variants of anthocyanin-related genes between cultivars results in tissue-specific accumulations of purple pigments. Thus, broad genetic variations of anthocyanin profile, and tissue-specific distributions in carrot tissues and organs, can be observed, and the ratio of acylated to non-acylated anthocyanins varies significantly in the purple carrot germplasm. Additionally, anthocyanins synthesis can also be influenced by a wide range of external factors, such as abiotic stressors and/or chemical elicitors, directly affecting the anthocyanin yield and stability potential in food and beverage applications. In this study, we critically review and discuss the current knowledge on anthocyanin diversity, genetics and the molecular mechanisms controlling anthocyanin accumulation in carrots. We also provide a view of the current knowledge gaps and advancement needs as regards developing and applying innovative molecular tools to improve the yield, product performance and stability of carrot anthocyanin for use as a natural food colorant.

## 1. Carrot as a Source of Anthocyanin for Natural Colorants

### 1.1. Application and Potential of Carrot as a Source of Natural Anthocyanins

The global market of natural colorants in the food industry was valued at USD 1.32 billion in 2015, and is expected to continue to grow over 7% annually between 2017 and 2022 [1]. This growth is due, in part, to the increased awareness of environmental hazards, the potential health benefits associated with the consumption of natural pigments, and the growing interest of the consumers in “natural” solutions [2]. To satisfy consumer demand, major food and beverage companies are moving towards replacing synthetic colors with plant-derived natural pigments in their products. However, the use of natural pigment is challenging due to interactions with food ingredients, the weaker tinctorial strength, the lower stability, and the challenges of matching the desired hues [3]. In addition, producing natural colorant is more expensive and requires efficient logistics. Indeed, while synthetic colorant is a more standardized industrial process, the production of natural colorants involves multiple steps, from development and production of the raw material, to extraction, dying and packing, which are more difficult to standardize. Comparisons of the synthetic vs. natural colorant prices are rarely reported in the scientific literature, but based on some food colorant industry experts, the cost of natural colors is about five times higher than synthetic colors on average, and in some cases (e.g., confectionery products) it can be as much as 20 times more expensive than synthetic (Peter Thorninger, https://www.nutritionaloutlook.com/view/switching-synthetic-natural-colors-here-are-your-challenges). Despite these challenges, the consumer demand for products that have a “clean label”, meaning they are free of artificial additives, continues to grow and represent the main driving factor for this shift [2,3]. For this reason, in order to achieve a sustainable natural colorant production system and market, it is becoming very critical to seek innovative solutions by taking advantage of the most advanced technologies.

Anthocyanin-rich extracts are currently among a group of commercially viable color ingredients, and are regulated as a color additive, although not defined as a “natural color” by the Food and Drug Administration (FDA). In the EU, water-based plant extracts containing anthocyanins within a natural range are widely permitted by the European Food Safety Authority (EFSA) for use as coloring in foodstuff due to their low toxicity [3], and are generally accepted as safe. However, when selectively extracted, further refined or concentrated, anthocyanins must be labeled with an E number (E163) as a color additive in the EU (Commission Directive 1333/2008 (EC).

Anthocyanins are a class of flavonoid antioxidants, and they represent some of the most vivid colors in nature, varying amongst shades of red, purple and blue, and they are produced in various plant tissues, including the leaves, roots, flowers and fruits. However, these natural pigments are generally unstable, and are susceptible to degradation driven by temperature, acidity and light exposure. In the past, this has limited their broad use as natural colorants. Recent advances in food technology and the identification of new sources of anthocyanins have contributed to a significant expansion of their use, especially in beverages. Anthocyanin’s share of the industry revenue has grown significantly over the last five-year period, reaching an estimated 9.5% share in 2018 [4]. Anthocyanins are likely to continue to be a key component of the “natural” food color industry, and remain a fast-growing category. The continued transition from artificial red, purple and blue color additives to anthocyanin-based pigments by food manufacturers will be driven by improvements in their chemical stability and versatility across broader food product forms.

In recent years, black or purple carrots have received much interest as natural sources of anthocyanins for application in the food industry (e.g., coloring soft drinks, fruit juices, jellies and confectioneries) [5]. Anthocyanin pigments extracted from purple carrot offer advantages over extraction from other fruit and vegetable sources due to the large concentration of anthocyanins in the former, which has been reported to be as much as 18 mg/100 g of fresh weight [6], and compared to other natural pigment sources (e.g., red cabbage) has low to no off-flavors. Additionally, a wide range of colors can be obtained from black carrot pigments, such as strawberry pink at low pH, versus purple and blue shades at higher pH [7,8]. This versatility is useful for coloring fruit juices, soft drinks and other food products [7], and could be used as a replacement for synthetic colorants such as FD&C Red 40 (allura red) [3,8,9]. Researchers investigating the color decay of ferric anthocyanin observed that during storage and thermal treatments, the pigment sources varied significantly; however, black carrot extract was the most promising pigment source of cobalt blue hues in pectin-stabilized solutions [10]. Compared to other anthocyanin-rich crops, like grapes, purple carrots contain lower amounts of non-anthocyanin phenolics that cause hazing and precipitation, and therefore carrots are a better source of anthocyanins [7]. Further, in a recent survey, 8 out of 10 consumers preferred seeing carrot extract on the food label to seeing synthetic colorant [11]. A review by Cortez et al. [12] highlighted that in the last five years, several processes for improving natural colorant stability using carrot extracts have been patented. New cultivars and methods for increasing anthocyanin production from carrot plants have also been developed and patented [13,14].

Acylated anthocyanins are an economically important colorant used in the food industry due to their increased stability at higher pH and their longer shelf-life [15]. In this regard, purple carrots also accumulate a high percentage of mono-acylated forms of anthocyanins [15,16], and unlike radish and red cabbage, the former do not require the removal of the sulfur aroma [15]. Acylation of anthocyanins is catalyzed by an acyltransferase [17], and acylation influences the cellular transport and stability of anthocyanins, which directly impacts their production, conservation, color, and thermal and shelf-life stability [15] when used as a natural dye or pigment [18,19]. Additionally, nonacylated anthocyanin are several times more bioavailable than acylated anthocyanin [20,21], and the consumption of carrot-derived anthocyanins may provide health benefits to the consumer [22]. For example, their high antioxidant capacity has been associated with protection against some forms of cancer [23,24], improved vision, reduced risk of diabetes [25], and enhanced cognitive and memory function [26]. Although there are no official statistics of worldwide purple carrot production, estimations by the food industry based on the annual need of 10,000 tons of carrot-derived anthocyanins place worldwide purple carrot production at approximately 100,000 ha [27].

### 1.2. Diversity for Anthocyanin Composition in the Purple Carrot Germplasm

**Figure 1:**
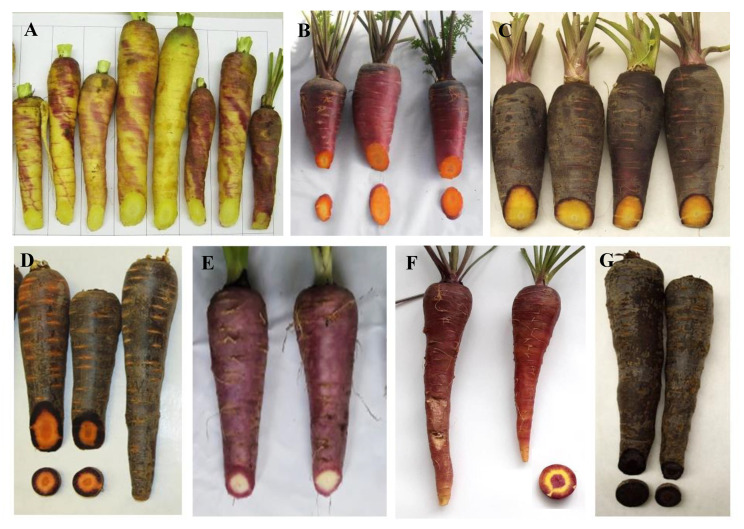
Examples of the extent of phenotypic variation of root anthocyanin pigmentation in the purple carrot germplasm (modified from Cavagnaro et al. [35]). The following phenotypes are illustrated: (**A**) carrot roots with non-uniform purple pigmentation in the root surface, in which purple pigmentation was visually estimated as the ‘percentage of the root surface covered with purple’, a trait called RTPE (root total pigment estimate) [33]); (**B**) presence of anthocyanins in the outermost epidermal layer; (**C**–**E**) anthocyanins in the root epidermis and cortex; (**F**) anthocyanins in the epidermis, cortex and xylem; (**G**) anthocyanin pigmentation in all root tissues (epidermis, cortex, phloem and xylem). Variation of anthocyanin pigmentation in the leaf petioles, as well as of root carotenoids (evidenced by the orange, yellow and white colors), can also be observed.

The purple carrot germplasm exhibits broad genetic diversity, with regards to the total anthocyanin concentration and the distribution of these pigments across different root tissues. To date, several studies have characterized anthocyanin pigments—by HPLC analysis—in more than 30 carrot lines, including accessions from germplasm banks and open-pollinated (OP) and hybrid commercial varieties, reporting an overall range for total anthocyanin content of 0.5–191 mg/100 g fresh weight (fw) [6,28,29,30]. In purple carrot roots, the total anthocyanin content is strongly and positively correlated with total phenolics content, with correlation values (r) ranging from 0.85 [30] to 0.99 [31], suggesting that anthocyanins represent a large fraction of the phenolics in purple carrots. It is noteworthy that the purple color intensity and the extent of the root tissues covered with purple are both directly associated with the total root anthocyanin [32]. Figure 1 depicts the range of root phenotypic variation that can be found in the purple carrot germplasm. In most purple-rooted genetic backgrounds, anthocyanins are mainly expressed in the outer epidermal layer of the root, but can also be expressed in the cortex (outer-phloem), phloem and xylem (core) tissues, presenting different pigmentation patterns across these tissues, to the extreme extent of having all the root section fully saturated with the purple pigment (Figure 1). Thus, carrot accessions with dark purple color throughout the entire root section tend to have the highest anthocyanin content, whereas those with purple pigmentation in only the outermost tissues usually have low levels of anthocyanins. In most of the purple genotype evaluated to date, root surface is fully and uniformly purple. However, in a few studies [33], the color of the root surface was reported to be not uniformly purple, and the purple color was estimated as a percentage of the root surface [33]. Visual root analysis performed to date has indicated that purple germplasm from Syria (e.g., Homs type) and China (e.g., Ping Ding type) accumulate anthocyanin in the outermost epidermal cell layer, cortex and/or xylem, but are not fully purple. Carrot roots with purple in all their tissue layers are typically from Turkey. According to Bannoud et al. [30,34], when both phloem and xylem tissues were pigmented, the total anthocyanin content in the phloem was higher than in the xylem. In addition to root pigmentation, anthocyanins can accumulate in other tissues and organs of the carrot plant, including the bracts of inflorescences, the flower petals, the seed, the leaf petiole and lamina, and the nodes and internodes of the flower stalk [32].

Variation in root anthocyanin composition can be found in the carrot germplasm. Purple carrots have, predominantly, five cyanidin glycosides, two of which are non-acylated and three are mono-acylated anthocyanins (Table 1) [6,16,29,30]. However, in some studies, traces of pelargonidin and peonidin have been reported in some genetic backgrounds [6,29,36]. Among the cyanidin glycosides, five major compounds, two non-acylated and three acylated, are commonly found in purple carrots (Table 1). The percentage of acylated anthocyanins (AA) relative to the total anthocyanin content found across different studies varied from 25% to 99% [6,23,28,29,33], although in most commercial cultivars, AA predominate over non-acylated anthocyanins (NAA), with the former generally representing more than 60% of the total anthocyanins. In terms of absolute concentration, up to 155 mg/100 g fw of AA and ~36 mg/100 g fw of NAA have been reported in purple carrot lines [16].

Anthocyanin profiles vary across carrot genetic backgrounds. The most abundant root pigments are cyanidin glycosides acylated with ferulic (Cy3XFGG), sinapic (Cy3XSGG) and coumaric acid (Cy3XCGG), with Cy3XFGG being the main pigment in most of the accessions evaluated to date [16,28]. Coincidently with these reports, anthocyanin profiles in 208 purple-rooted carrots from an F_2_ mapping population generally revealed Cy3XFGG as the predominant pigment, followed by Cy3XSGG, representing on average 49% and 23% of the total anthocyanin content, respectively [33]. Similar results were found recently in an F_2_ population used for mapping QTL as conditioning root tissue-specific anthocyanins, with Cy3XFGG being the major pigment in both the phloem (accounting for 31% of total anthocyanins) and xylem (~52% of total anthocyanins), followed by Cy3XSGG, accounting for 23% and 34% of the total anthocyanins in these respective tissues, respectively. Among the non-acylated pigments, Cy3XG is generally found at a higher concentration than Cy3XGG, as observed in most commercial cultivars and accessions from germplasm banks [6,16,29,30], as well as in F_2_ and F_4_ populations recently characterized [34]. The opposite trend (i.e., Cy3XGG > Cy3XG) was found in another F_2_ previously described [33].

Characterizing the extent of the genetic and compositional variation in purple carrots is important from a breeding point of view, for both the production of chemically stable food dyes (e.g., aiming at developing cultivars with high concentrations of AA) and for the fresh market. Although purple germplasms have been previously characterized with regards to their anthocyanin composition, little information has been published to date on the evaluation of genetic diversity in these materials. In an early study, a large dataset of 4000 SNP markers was used to investigate carrot structure and domestication in a collection of 84 cultivated and wild carrot accessions from diverse geographical origins, including 9 purple-rooted accessions from Middle East and Central Asia [37]. The study revealed that all the purple carrots included in the study were genetically distinct, and they clustered with Eastern-cultivated carrots of other colors, clearly separating them from Western-cultivated and wild carrots from various origins [37]. More recently, Ipek, et al. [38] used 20 SSR markers to estimate genetic diversity in purple carrot populations of Ereğli, Turkey, composed of open-pollinated local cultivars and landraces. Substantial molecular variation in the SSR loci was found among these germplasms (i.e., 18 polymorphic SSRs yielded 106 alleles, and polymorphism information content (PIC) ranged from 0.29 to 0.85, with the expected heterozygosity ranging from 0.32 to 0.87), indicating a broad genetic diversity in these Turkish carrot populations. Although no pigment characterization was reported for these materials, these genetic resources are potentially valuable for the development of new purple carrot cultivars. Additional studies in other purple carrot germplasms, which combine both pigment compositional and molecular marker analyses, are necessary for a more detailed characterization of these materials, and to understand the origins of the purple root phenotypes from different genetic backgrounds. From a breeding perspective, the characterization of the purple carrot germplasms collected, based on their root anthocyanin content and AA:NAA ratio, will allow the selection of materials with high anthocyanin concentrations and specific pigment profiles, for the development of new varieties for either fresh consumption or the extraction of food colorants.

### 1.3. Shelf-Life Stability of Anthocyanins Derived from Carrot (pH, Temperature and Shelf-Life)

The shelf-life of naturally pigmented food products is, in general, reduced as compared to that of synthetic colorants, which results in expired and wasted food products. Variation in pH, temperature and light, as well as complexing with other compounds, can influence the stability of natural anthocyanin pigments during storage, and result in a modification or fading of the food color [39,40]. Acylated anthocyanins, as opposed to non-acylated anthocyanins, are the predominant anthocyanin-based colorants used by the food industry due to their greater color stability at a higher pH [15]. Acylation influences the cellular transport and stability of anthocyanins, directly influencing their accumulation and shelf-life conservation, when used as a natural dye or pigment [18,19]. Research into the stability of black carrot anthocyanins, regarding pH, temperature and shelf-life, has been thoroughly investigated [41]. Here we provide a summary of these studies, with an emphasis on the effect of the anthocyanin structure and carrot chemical composition on anthocyanin stability (Table 2). Storage at lower temperatures resulted in slower anthocyanin degradation as compared to storage at higher temperatures [42,43,44,45,46,47]. No significant effects on anthocyanin degradation were detected at temperatures below 4 °C and/or subfreezing [48,49]. At all temperatures, the NAA were significantly less stable than the AA [42,46,47]. For example, after storage at 40 °C, Turker et al. [42] reported that AA retained between 7.9% and 48.9% of their initial level, whereas NAA retained only 0% to 11% of their initial level. Compared to blackberry and acai anthocyanins, the rate of degradation of carrot anthocyanin has been shown to be slower [48].

The effect of pH has also been investigated in multiple studies [6,8,44]. In general, degradation rates of anthocyanin increased with pH, especially above 5. Montilla et al. [6] observed that Cy3XSGG exhibited a lower visual detection threshold at pH 3–5 than Cy3FGG and Cy3XCGG, suggesting that Cy3XSGG was more stable. In addition, a higher solid content was associated with a higher degradation of anthocyanins during thermal treatment (heating).

Comparing results from multiple studies, Kirca et al. [44] observed that anthocyanins from black carrot have greater stability during storage than those from blood orange, sour cherry, red-fleshed potato, red radish and blackcurrant during storage. Altogether, studies suggest that AA were significantly more stable than NAA at all storage temperature and pH ranges evaluated [41,42,46,50,51]. Furthermore, the high content of AA present in some black carrot genotypes has been suggested to be the key factor in explaining the higher stability of carrot anthocyanin as compared to other natural sources of anthocyanin.

## 2. Genetics and Genes Controlling Anthocyanin Pigmentation in Carrot

### 2.1. Anthocyanin Genetics in Carrot

**Figure 2:**
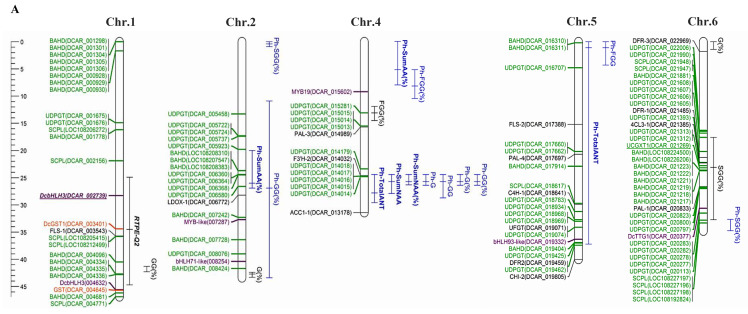

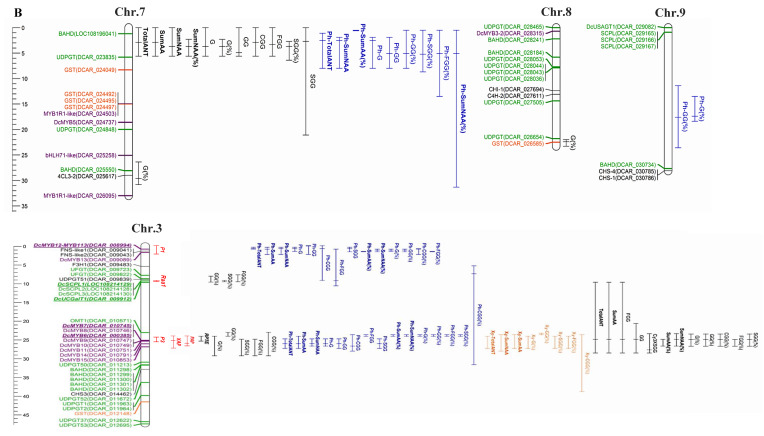

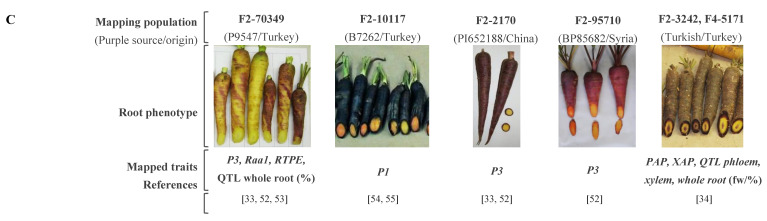
Genomic locations of anthocyanin QTLs and phenotypes mapped in carrot with respective references [33,34,52,53,54,55]. (**A**,**B**) Representative QTL and simply-inherited traits conditioning the presence and concentration of anthocyanins mapped onto carrot chromosomes 1–2, 4–9 (**A**) and 3 (**B**), displaying the physical position of genes associated with anthocyanin biosynthesis. Regulatory genes (*MYB*, *bHLH* and *WD40*) are indicated in purple font, structural genes in black, anthocyanin-modifying genes (i.e., acyltransferases, glucosyltransferases, methyltransferases) in green, and genes involved in intracellular transport of anthocyanins in orange. For regulatory genes, only those clustering with functionally characterized regulatory genes from other species—in orthology and/or phylogenetic analyses—are included. The genes are labeled by name followed by the DCAR or LOC number, in parenthesis. The physical position of each gene in the chromosomes is expressed in terms of nucleotide coordinates from the carrot genome assembly [56], and indicated by the ruler on the left of each group of chromosomes (units are in Mb). Simply-inherited phenotypic traits are indicated in red, italic and bold. QTL conditioning absolute (i.e., expressed on a fresh weight basis) or relative pigment concentration (i.e., % of the total anthocyanin content) in the whole root (in black), as well as in the root phloem (in blue) or xylem tissues (in orange), were mapped. QTL for total or combined anthocyanin pigments (e.g., sum of acylated anthocyanins) are indicated in bold. QTL bars indicate the 1.5 LOD interval (nt) and the position of the maximum LOD value. QTL are labelled by their pigment abbreviations G—Cy3XG, GG—Cy3XGG; CGG—Cy3XCGG; FGG—Cy3XFGG; SGG—Cy3XSGG; TotalANT—total anthocyanins; SumAA—sum of acylated anthocyanins (i.e., CGG+FGG+SGG); SumNAA = sum of non-acylated anthocyanins (i.e., G+GG) preceded by the type of root tissue (Ph—phloem, Xy—xylem), in the case of tissue-specific QTL, and followed by “(%)” to indicate QTL expressed as relative concentration. Redundant traits and QTL that have been mapped in other carrot genetic backgrounds with similar results, as well QTL identified with alternative methods of analysis, were not mapped herein. Further information on these and other QTL not included in this figure is presented in Supplementary Tables S1–S3. (**C)**. Main characteristics of the segregating populations and purple-rooted sources used for mapping anthocyanin traits.

Important advances in the understanding of the genetic control of carrot anthocyanin pigmentation have been made. A summary of all mapped anthocyanin phenotypes and QTLs is reported in Supplementary Tables S1–S3, and is illustrated in Figure 2. In total, 158 loci or QTLs associated with anthocyanin accumulation in root or petioles were mapped for carrots. Among the different anthocyanidins detected in carrots, QTLs analyses were performed only for the cyanidin derivatives, since poenidin and perlargonidin derivatives were detected at a very low concentration.

A first study by Simon [57] described that a simply-inherited locus, called *P*_1_, controlled purple pigmentation in carrot roots, whereas another major locus (*P*_2_) conditioned pigmentation in the nodes, with *P*_1_ and *P*_2_ being genetically linked at ~36 cM. Subsequent studies were done by Vivek and Simon [54], and Yildiz et al. [55], into the Turkish ‘B7262’ genetic background, which presents purple pigmentation only in the outer root tissues (periderm and outer phloem) and has green petioles. Another dominant locus, called *P*_3_, conditioning anthocyanin accumulation in the root periderm and petioles of a Turkish (P9547) and a Chinese (PI652188) carrot lines, was later described and mapped to chromosome 3 [33]. Comparative analysis, segregating families derived from the B7262, P9547 and PI652188 backgrounds, revealed that *P*_1_ and *P*_3_ correspond to different loci in chromosome 3, and that the two loci are more than 30 cM apart [33].

In subsequent studies, segregation for root and petiole pigmentation was investigated and mapped to different genetic backgrounds, including an F_2_ developed from a Syrian purple carrot (BP85682), and advanced generations (F_3_, F_5_) of the mapping populations used previously by Cavagnaro et al. [33] and Iorizzo et al. [52]. Purple pigmentation in the root periderm and leaf petioles was fully co-segregated, and this suggested a single dominant gene for the genetic control of both traits. Comparative linkage analysis, with other populations harboring previously reported loci conditioning anthocyanin pigmentation, demonstrated *P*_3_-conditioned purple pigmentation in the Syrian background BP85682 as well. In another population, namely 5723, purple pigmentation in the petioles also segregated according to a 3:1 ratio, consistent with a single gene model, and this trait was mapped into the same region of *P*_3_. Thus, in some genetic backgrounds, *P*_3_ controls both root and petiole anthocyanin pigmentation, whereas in other backgrounds, purple petiole is independent of purple root. In B7262, ‘root pigmentation’ is conditioned by the *P*_1_ locus. This result suggested that within the *P_3_* region, the loci controlling purple petioles and roots are tightly linked, but can act independently. Further, based on the extensive expansion of knowledge achieved in recent years regarding anthocyanin genetics in carrots, we here hypothesize that the control of the purple node, which was originally ascribed to *P*_2_ by Simon [57], actually corresponds to the *P*_3_ locus, which controls the purple petiole as mapped in the 5723 population. In addition to *P*_1_, *P*_2_ and *P*_3_, a simply-inherited trait, called Raa1 for ‘root anthocyanin acylation’, conditioning the percentage (%) of AA versus NAA, that is, the high % of AA being dominant over low % AA, was described and located—by linkage mapping—in chromosome 3, with its position being 17.9 cM from *P*_3_ [33]. Furthermore, very recently, Bannoud et al. [34] described and mapped two simply-inherited loci controlling the presence/absence of purple pigmentation in the root xylem and phloem, with purple being dominant over non-purple. These loci, called *XAP* and *Phloem*, for ‘xylem and phloem anthocyanin pigmentation’, were mapped in the same chromosome region of *P*_3_, together with another major locus controlling the presence/absence of pigmentation in the petioles, named *PAP* for ‘petiole anthocyanin pigmentation’ [34].

In addition to the simply-inherited loci controlling the presence or absence of anthocyanins in different root and leaf tissues, several quantitative trait loci (QTL) conditioning the concentration of root anthocyanins (Cy3XG, Cy3XGG, Cy3XFGG, Cy3XSGG and Cy3XCGG) have been described and mapped in the last six years [33,34,53,58]. In a first study, Cavagnaro et al. [33] mapped 15 QTL controlling the concentrations of four individual anthocyanin pigments (Cy3XG, Cy3XGG, Cy3XFGG and Cy3XSGG) as well as the total root anthocyanins (‘RTPE’, for ‘root total pigment estimate’) in an F_2_ family, named 70349, developed from the Turkish purple root source P9547. Segregation analysis for purple vs. non-purple root in 70349 and its F_3_ derivative populations indicated that two dominant loci interact epistatically in the genetic control of purple root pigmentation. The 15 QTLs were mapped to chromosomes 1, 2, 3, 6 and 8, and eight of them with the largest effects (26.6–73.3%) were co-localized to two regions of Chromosome 3. In the *P_3_* region, the co-localization of a major QTL for *RTPE* (RTPE-Q1), which explained 50.5% of the variation, and QTLs for four root anthocyanins were found. A second QTL for *RTPE* (RTPE-Q2) explaining ~5% of the variation with lower phenotypic effect (~5%) was identified in Chromosome 1 (Figure 2). RTPE-Q1 and -Q2 explained the two-gene model observed for root purple color segregation in F_2_–F_3_ families. However, QTL interaction analysis indicated that RTPE-Q1 has a dominant effect, and is required for the expression of the RTPE phenotype. These results confirmed that the *P_3_* region, where RTPE-Q1 was mapped, plays a key role in the expression of anthocyanin in carrot roots and petioles, and highlighted that a second QTL region (RTPE-Q2) mapped in chromosme 1 also influences—to a lesser extent—the total anthocyanin concentration in the carrot root.

In a recent study by Iorizzo et al. [52], high resolution mapping was performed for *P_3_* using a larger population size of the same genetic background used by Cavagnaro et al. [33] (*N* = 187), reporting the identification and mapping of the same major QTL (Figure 2). A substantially smaller map region was attained for RTPE-Q1 and other root anthocyanin QTLs in this new map (Supplementary Table S2), as a consequence of the higher map resolution. Thus, in this region, Cavagnaro et al. [33] reported five overlapping QTL within a 12 cM region, whereas in the new map they spanned 6.3 cM, with co-localized QTLs for *RTPE* and three anthocyanin pigments within a 3 cM region. 

In the study by Cavagnaro et al. [33], co-localized QTLs for the root AA Cy3XSGG and Cy3XFGG, and the NAA Cy3XGG, were also found in a small map region (3.6 cM) of Chromosome 3, and they all co-localized with *Raa1*. The QTL for Cy3XGG, which is proposed as the most likely substrate for acylation, had the highest LOD value (104.7), the largest phenotypic effect (73.3%) and the shortest confidence interval (0.7 cM) of all the 15 mapped QTL. These data suggest that *Raa1* controls the ‘high’ versus ‘low’ percentage of acylated anthocyanins in carrot roots. Because the acylation of anthocyanins influences bioavailability [20,21] and pigment stability [59,60] understanding the genetic basis of anthocyanin acylation may be important for carrot breeding programs aimed at developing new cultivars with high levels of chemically-stable acylated pigments. Very recently, the *Raa1* locus was characterized in detail by Curaba et al. [53] (described in Section 2.2).

In a newer study, Bannoud et al. [34] used two mapping populations (3242 and 5171) to map QTLs associated with total anthocyanin content, individual anthocyanin (Cy3XG, Cy3XGG, Cy3XFGG, Cy3XSGG and Cy3XCGG) content and relative percentages of individual anthocyanin, in the root phloem and xylem. In these two populations, anthocyanin accumulations in the phloem and outer-phloem (cortex) were not always distinguishable, and the purple pigmentation in these tissue layers was scored as phloem-specific (Cavagnaro personal communication). In total, 150 QTLs across seven chromosomes were mapped, with 8 of these QTLs associated with anthocyanin accumulation in the xylem, and 95 of the QTLs were mapped in chromosome 3 (Supplementary Tables S1–S3). Out of these 95 QTLs, 52 overlapping with the *P*_1_ region were associated with anthocyanin accumulation in the phloem, and 43 overlapping with the *P*_3_ region were associated with anthocyanin accumulation in the phloem and xylem. The other 24 and 12 QTLs associated with anthocyanin accumulation in the phloem mapped to two overlapping regions on chromosome 4 and 7, respectively.

Overall, across all genetic studies for anthocyanin pigmentation in carrots, three QTL regions (*P*_1_, *P*_3_ and RTPE-Q-2) control the presence of purple pigmentation in the carrot root in a tissue-specific manner, one QTL region controls purple pigmentation in the petiole (*P*_3_), and another QTL region (*Raa1*) controls anthocyanin acylation. Across all studies, the *P*_3_ region has been identified as a candidate region harboring the key gene(s) controlling anthocyanin accumulation across all tissues, while the *P*_1_ region is involved in the regulation of anthocyanin accumulation in the root out-phloem and/or phloem of specific genetic backgrounds. Several other QTLs with lower effects were identified, and will serve as a foundation for studying the overall molecular mechanisms and their interactions controlling anthocyanin synthesis, storage and degradation. Given the tissue-specific nature of anthocyanin accumulation in carrot, by dissecting the genes involved in tissue-specific anthocyanin expression, breeders will gain a better understanding of the genetics underlying these traits, and may be able to predict root color phenotypes in directed crosses. DNA markers associated with anthocyanin accumulation in carrots have been identified, and the regulatory and structural genes involved in the anthocyanin biosynthetic pathway are being investigated [56,58].

### 2.2. Anthocyanin Structural Genes

**Figure 3:**
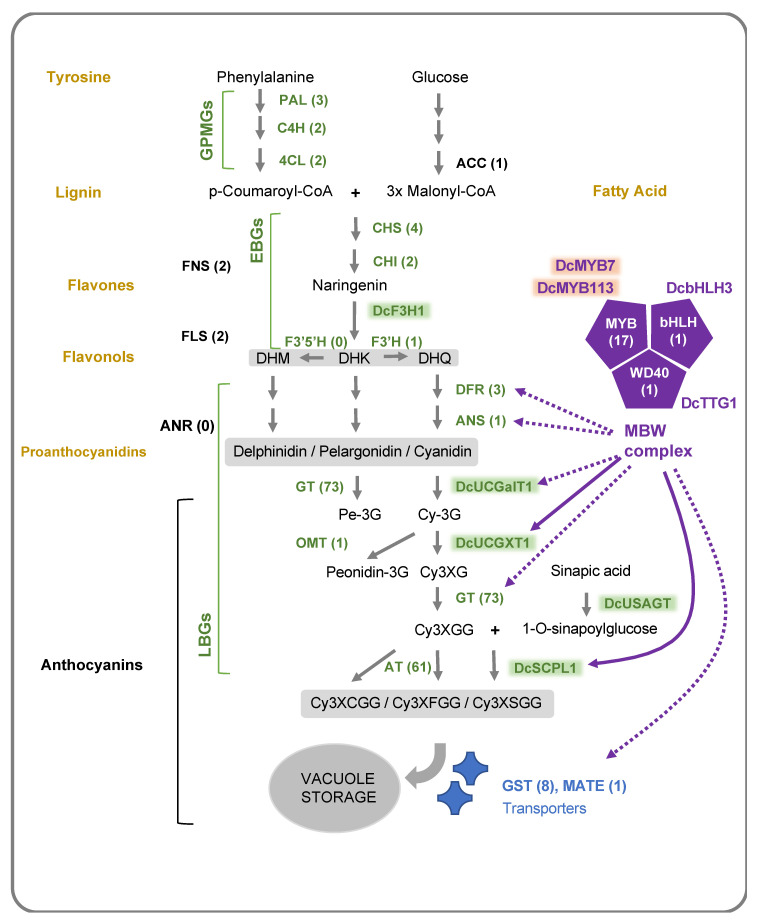
Anthocyanin biosynthetic pathway in the carrot. Structural enzymes of the phenylpropanoid pathway participating in the formation of anthocyanins are in green: PAL, phenylalanine ammonia lyase; C4H, cinnamate 4-hydroxylase; 4CL, 4-coumaroyl (CoA-ligase); CHS, chalcone synthase; CHI, chalcone isomerase; F3H1, flavanone 3-hydroxylase 1; F3′H, flavonoid 3′-hydroxylase; F3′5′H, flavonoid 3′,5′-hydroxylase; DFR, dihydroflavonol reductase; ANS, anthocyanidin synthase; GT, glycosyltransferase; AT, acyltransferase; MT, methyltransferase. Enzymes involved in anthocyanin transport are in blue: GST, glutathione S-transferase; MATE, multi-antimicrobial extrusion. Regulatory enzymes composing theMYB-bHLH-WD40 (MBW) complex are in purple and their regulations of anthocyanin-related genes are indicated by purple solid and dashed arrows when the regulation is confirmed or potential, respectively. Other metabolic enzymes known to influence anthocyanin accumulation are in black: FNS, flavone synthase; FLS, flavonol synthase; ANR, anthocyanidin reductase; ACC, Acetyl-CoA carboxylase. Related branches of competing pathways leading to the production of non-anthocyanin compounds are indicated in yellow. For each enzyme family, the number of corresponding coding genes identified in carrots (from Supplementary Table S4) is indicated in the brackets (for MBW enzymes, only the number of coding genes orthologous to anthocyanin-related genes from other species in indicated). Functionally characterized enzymes are highlighted, and DcbHLH3 and DcTTG1 are indicated as the best candidates, but they were not functionally validated in planta. Abbreviations—dihydroflavonols: DHM, dihydromyricetin; DHK, dihydrokaempferol; DHQ, dihydroquercetin.

Anthocyanin accumulation is determined by the activity of structural genes, which are divided into general phenylpropanoid metabolism genes (abbreviated here GPMGs) and early and late biosynthesis genes (EBGs and LBGs, respectively) [61,62]) (Figure 3). GPMGs are required for the synthesis of other phenylpropanoids, such as lignin and Acetyl-CoA carboxylase (ACC), for the production of fatty acid compounds containing 4-coumaroyl-CoA and malonyl-CoA, respectively [61,63]. EBGs are shared for the biosynthesis of multiple flavonoids, whereas LBGs are more specific to the anthocyanins [63,64]. LBGs include genes coding for modification enzymes, such as glycosyltransferases (GTs) and acyltransferases (ATs), which catalyze the addition of sugar moieties and acyl groups, respectively, resulting in specific decoration patterns that greatly influence their function and stability. Since the carrot genome was released in 2016, curated annotation identified 159 potential structural anthocyanin genes, either located within an anthocyanin-related QTL or differentially expressed between purple and non-purple tissues [34,53,56,65,66,67] (Supplementary Table S4). These include 8 GPMGs, 8 EBGs and 139 LBGs, including 73 GTs genes, 61 ATs genes and 1 O-methyltransferase (OMT) gene, coding for enzymes involved in anthocyanin glycosylation, acylation and methylation, respectively (Supplementary Table S4). Comparative analysis with other genomes, like grapevine and *Arabidopsis*, indicated that the carrot genome lacks the *Flavonoid 3’5’ hydroxylase* (*F3′5′H*) and the *anthocyanidin reductase* (*ANR*) genes. The ANR enzyme catalyzes the first step of the proanthocyanindin (PA) pathway (Figure 3), and *F3′5′H* is required to direct the flux toward the anthocyanin delphinidin derivatives, which perhaps partially explains the low diversity of the anthocyanin and flavonoid derivatives detected in carrots. The characterization of genes retained after three whole genome duplications (WGD) indicated that several flavonoid/anthocyanin genes are duplicated. For example, three copies of the phenylalanine ammonia-lyase (*DcPAL1*, *DcPAL3* and *DcPAL4*) were retained after each of the three WGDs [56]. Several ATs and GTs were organized in tandem clusters [53], likely as a result of recent tandem duplications [68,69]. Although the role of these duplicated genes in the carrot is still unknown, each of these duplicated genes may have acquired a specialized function in the expression of the pathway in specific tissues, or under specific environmental conditions (e.g., abiotic stresses).

The expression data from eight independent studies are available for 105 anthocyanin structural genes, including 90 with a detectable level of mRNA above a 1 RPKM threshold [34,52,53,55,67,70,71,72] (Supplementary Table S4). In this review, we integrated all these data so as to highlight differences and consistency. Differential gene expression analysis of purple vs. non-purple tissues identified 78 genes as being up or downregulated in at least one genotype, 54 of which were found to be upregulated in at least one purple root sample, including 7 genes (*DcPAL4*, *DcC4H1*, *DcCHS1*, *DcCHI1*, *DcF3H1*, *DcF3’H1* and *DcDFR1*) consistently reported to be upregulated in at least nine independent carrot lines (Table 3). This may indicate that the transcriptional regulation of these seven genes was targeted early in the evolution of purple carrots, and may be well conserved among the various carrot cultivars used in breeding today.

The expression level in the petiole, which was only measured in one study, revealed that five glycosyltransferases coding genes (*DcUDPGT1*, *8*, *32*, *50* and *70*) and *DcFLS2* could be specifically active in the aerial parts [52] (Supplementary Table S4). The downregulation of *DcFLS2* in purple pigmented petiole, associated with the upregulation of *DcDFR1*, could significantly contribute to directing the metabolic flux of dihydroflavonols toward the anthocyanin pathway in this tissue, as it was observed in the corolla of other plant species [73,74]. The highest level of anthocyanin accumulation could be achieved when both genes were antagonistically regulated [75]. Similarly, two flavone synthase like genes (*DcFNS-like1* and *DcFNS-like2*) and two flavanone hydroxylases (*DcF3H* and *DcF3′H*), which compete for Naringenin as substrate, present opposite expression patterns in dark vs. pale purple phloem [34]. Increasing the level of *FNS* in transgenic celery dramatically reduces the anthocyanin content, as well as the expression levels of *F3′H* and *DFR*, suggesting the existence of a molecular mechanism coordinating their expression in the Apiaceae family [76]. Reducing FNS activity in black-colored dahlia plants was also linked to the accumulation of high amounts of anthocyanins [77]. Interestingly, however, a minimum level of FNS activity is needed to produce flavone co-pigments which could help in stabilizing the accumulation of anthocyanins [78]. The transcriptional regulation of genes coding for metabolic branching point enzymes, such as FNS/F3H and FLS/DFR, plays a critical role in balancing the metabolic flux of phenylpropanoids, and is likely to be a determinant factor in the production of purple pigments in carrot [74,79,80,81]. Among all the structural genes tested, *DcDFR1* is the only one that is always found upregulated in all purple tissue, making it the most reliable marker for anthocyanin biosynthesis and a possible bottleneck of the pathway in carrot. Indeed, increases in *DcDFR1* expression tend to be proportional to the accumulation levels of anthocyanins observed in a carrot population that segregated for purple color intensity in the root phloem [34].

Among the annotated carrot anthocyanin structural genes, five were functionally characterized: *DcF3H1* [83], *DcUCGalT1* [65], *DcUCGXT1* [72], *DcSCPL1* [72] and *DcUSAGT* [66] (Figure 3, Table 3 and Supplementary Table S4). The knockout of *DcF3H1* (*DCAR_009483*) using the CRISP/Cas9 system caused the discoloration of calli, which validated the function of this gene in the biosynthesis of anthocyanin in carrot, as well as demonstrating the successful application of CRISPR/Cas9 in carrots. *DcUCGalT1* (*DCAR_009912*) has been shown to catalyze the formation of cyanidin 3-galactoside (Cy3G) in vitro, and *DcUSAGT* (*DCAR_029082*) was shown to catalyze the transfer of a glucose moiety to the carboxyl group of sinapic acid, thereby forming 1-O-sinapoylglucose [65,66]. 1-O-sinapoylglucose serves as an acyl donor in the acylation of cyanidin-3-(2″-xylose-6-glucose-galactoside) (Cy3XGG) into its acylated counterpart, cyanidin-3-(2″-xylose-6″-sinapoyl-glucose-galactoside) (Cy3XSGG), which helps stabilize the accumulation of anthocyanins in purple carrots. This reaction was recently attributed to DcSCPL1 (also named DcSAT1) by two independent studies [53,72]. *DcSCPL1* was first identified as a strong candidate for the *Root Anthocyanin Acylation 1 (Raa1)* locus controlling the formation of Cy3XSGG and Cy3XFGG in the carrot storage roots of three mapping populations [53]. Sequence analysis of *DcSCPL1*, in both high and low acylated backgrounds, revealed the presence of two distinct alleles, one functional and the other not, that can easily be identified by PCR. Another study by Xu et al. [72] supported this hypothesis by showing that overexpressing *DcSCPL1* in the calli of the dark purple-rooted carrot ‘Deep purple (DPP)’ increases the production of Cy3XSGG [72].

In this review, we localized the physical location and boundaries of all published QTLs, and annotated the anthocyanin structural genes identified in carrots to date, noting that multiple LBGs and EBGs were localized within anthocyanin QTLs (Table 3; Supplementary Table S4). This includes, for example, a cluster of four *DcSCPL-ATs* (*LOC108227197*, *LOC108227196*, *LOC108227198* and *LOC108192824*), of which three are upregulated in purple roots, and are co-localized with two QTLs for Cy3XSGG, mapped in chromosome 6 [34]. Interestingly, Curaba et al. [53] noted that two of these genes, *LOC108192824* and *LOC108227198*, clustered with *DcSCPL1* in clade IA-1, and possess the predicted functional SCPL domain/motifs. One *BADH-AT* (*LOC108196041*) co-localized with 15 QTLs on chromosome 7, of which 9 were QTLs associated with a potential acylation function (Table 3; Supplementary Table S4). Furthermore, a cluster of 5 *LBG-GTs* and *DcF3’H1* overlapped with 12 phloem-specific QTLs located in chromosome 4, which include a QTL for total anthocyanin [34]. Interestingly, in multiple studies, *DcF3′H1* is found to be upregulated in purple carrot roots, and is highly co-expressed with *DcMYB113* [72], a transcription factor controlling anthocyanin accumulation in the periderm and phloem (considered as outer-phloem in this review) (Supplementary Table S4). This is the first time that these published results have been integrated, and therefore these new findings provide new opportunities to further investigate the genetic pathway controlling the synthesis and various decoration patterns of anthocyanins present in carrots, which likely involves multiple *AT* and *GT* genes coding for specialized enzymes with different substrate specificities.

Overall, across all the published anthocyanin genetic studies in carrots, none of the chromosome locations harboring GPMGs or EBGs overlapped with any of the major QTL regions (*P*_1_, *P*_3_, *RTPE-Q-2*, *XAP*, *PAP* or *Phloem*) controlling the expression of anthocyanins in purple root tissues and petioles. The expression levels of most structural genes related to flavonoid metabolic pathways are consistently higher in purple vs. non-purple tissues. Duplicated genes appear to have divergent expressions of root tissue-specificity, such as *DcCHS1*, which is upregulated mostly in purple xylem, compared to *DcCHS2* and *DcCHS9*, which are upregulated mostly in purple phloem tissues, or *DcDFR1*, which is upregulated in purple xylem as opposed to *DcDFR2* and *3*, two genes that are slightly downregulated in the same tissue [67] (Supplementary Table S4). These results suggest that the purple phenotype is likely controlled by more than one of the transcription factors that control anthocyanin accumulation in carrot root and petioles by coordinating the expression of structural genes in a tissue-specific manner.

### 2.3. Regulatory Anthocyanin Genes

The regulation of the expression of anthocyanin structural genes, especially LBGs, is coordinated by the MYB-bHLH-WD40 (MBW) protein complex, in which the role of the MYB and bHLH transcription factors is critical to triggering anthocyanin accumulation in specific tissues [62,63,84,85,86,87] (Figure 3). The regulation of LBG activity by the MBW complex is well conserved among land plants, and several studies report the functional characterization of MBW members through transgenic expression in orthologous system [61,62,72,88,89,90,91,92,93,94]. Among 891 MYB-, bHLH- and WD40-coding genes mapped in the carrot genome by Iorizzo et al. (2019), 73 genes are potentially related to the anthocyanin metabolism through one of the following three criteria: overlapping with an anthocyanin-related QTL, differentially expressed between purple and non-purple tissues, or orthologous to a known anthocyanin-related gene from another species (Supplementary Table S4) [34,52,67,71,72,95]. Only 12 of them, including 11 anthocyanin-related *MYBs* (*A-MYBs*) and 1 anthocyanin-related *bHLH* (*A-bHLH*), *DcbHLH3*, possessed all three criteria, and therefore represent primary candidates for further investigating the regulation of purple pigmentation in carrot (Table 3). RNAseq data from specific tissue layers offers valuable information in order to identify the candidate genes controlling anthocyanin accumulation in the storage root. However, to date only a few studies have performed comparative transcriptome analysis by sampling specific root tissues. This is due in part to the limited knowledge that the carrot breeding and genetic community has regarding the genetic inheritance of anthocyanin accumulation in the different tissues, and also to an often inconsistent identification/naming of the different purple root tissues sampled/analyzed across studies. Considering that the most striking differences in purple pigmentation between carrot cultivars are between xylem and phloem tissues, it is interesting to notice that 10 *A-MYBs* and 14 *A-bHLHs*, including *bHLH3*, present a differential regulation between these two tissues (Supplementary Table S4). A-MYBs in other plant species were identified as either activators or repressors of the anthocyanin pathway [86,96,97]. Transcriptome data of carrots revealed that 4 *A-MYBs*, including 2 that were predicted as MYB1R1-like transcription factors (*DcMYB1R1-1* and *DcMYB1R1-2*), were downregulated in both phloem and xylem tissues, and may negatively regulate anthocyanin biosynthesis [67] (Table 3; Supplementary Table S4). Such repressors could inhibit the accumulation of anthocyanin by directly repressing the expression of structural genes, interfering with the MBW activity, or promoting the expression of competing enzymes that use the same substrates required for anthocyanin production [97]. Examples can be found in other plant species, such as in strawberries, wherein FaMYB1 can interact with bHLH proteins and repress the expression of structural genes at the lower end of the flavonoid pathway (ANS and GT) [94], or in the *Mimulus lewisii* flower, wherein *LAR1* represses the biosynthesis of anthocyanin by activating the expression of *FLS* [73].

Using a fine mapping approach, Iorizzo et al. [52] identified a cluster of six genes coding for A-MYB transcription factors (*DcMYB6, 7, 8, 9, 10* and *11*) in the *P_3_* region harboring overlapping QTLs (Table 3; Supplementary Table S4). The functional characterization of *DcMYB6*, using non-endogenous 35S promoter, demonstrated its ability to induce anthocyanin expression in *Arabidopsis*, but not in orange Kurodagosun (KRD) carrots [71,90]. The expression data indicate a genotype-specific regulatory activity for *DcMYB6*. While highly expressed in most purple root cultivars, *DcMYB6* expression does not correlate with anthocyanin pigmentation in all the purple-rooted carrot lines tested, and remains expressed in some non-purple tissues [34,52,71]. Within the *A-MYB* cluster, *DcMYB7* is the only gene specifically overexpressed in all purple vs. non-purple root tissues, and its expression was also detected in the purple petiole of two carrot lines [52,71] (Table 3; Supplementary Table S4). Further studies from the transgenic approach, using both overexpression and knockout carrot lines, showed that DcMYB7 is functional in at least three purple cultivars, and is essential for the production of purple pigments in at least one of them (DPP), making it the best candidate gene for the *P_3_* locus controlling anthocyanin pigmentation in the carrot storage root [71,98]. *DcMYB11* is the only *A-MYB* specifically expressed in all purple petiole, and represents the best candidate for the genetic control of petiole purple pigmentation (Table 3; Supplementary Table S4). More recently, another MYB transcription factor, *DcMYB113*, was identified as a candidate for *P*_1_, and was functionally characterized [72]. The expression of *DcMYB113* appears to be cultivar-specific, and restricted to the root periderm and phloem of the carrot cultivar ‘purple haze’ (PPHZ) (Table 3; Supplementary Table S4).

By identifying the key *A-MYB* regulatory gene(s), it may be possible to initiate and control the entire anthocyanin pathway in carrots. Indeed, the overexpression of either *DcMYB7* or *DcMYB113* in orange KRD cultivar triggers the accumulation of anthocyanins in the entire carrot tap root and petiole, although only *DcMYB7* under the control of a strong 35S-promoter was reported to induce purple pigmentation in the reproductive organs of the transgenic lines [71,72]. Both A-MYBs can interact with DcbHLH3, and could directly activate the expression of two structural genes related to anthocyanin structural modification—a glycosyltransferase, *DcUCGXT1* and an acyltransferase, *DcSCPL1* [71,72]. *DcbHLH3* co-localize with the RTPE-Q2 in chromosome 1, one of two major QTLs controlling anthocyanin accumulation in a mapping population derived from a Turkish carrot used as the purple root source progenitor [33]. Interestingly, the overexpression of *DcMYB113* in KRD leads to an increase in the Cy3FGG/Cy3SGG ratio, whereas the overexpression of *DcMYB7* in the same cultivar may have the opposite effects [71,72]. The differential activity of A-MYBs could be responsible not only for variations in the levels of anthocyanins being produced, but also their profile.

Among the five annotated carrot anthocyanin-related *WD40* (*A-WD40*) genes, only *DcTTG1* transcripts have been detected in carrot roots so far [52,95]. DcTTG1 was identified by Kodama et al., on the basis of its homology via Blast analysis to *Arabidopsis* TTG1. Here, we confirmed by orthologous, phylogenetic and synteny analysis that DcTTG1 cluster with TTG1 (Table 3; Supplementary Table S4). *TTG1* is constitutively expressed in all major organs, and is a constant member of the MBW complex required for the activation of the anthocyanin pathway and the determination of epidermal cell fate in *Arabidopsis* [99,100,101]. In carrots, *DcTTG1* is located in chromosome 6, and its location does not overlap with any anthocyanin-related QTLs previously mapped in carrots (Table 3; Supplementary Table S4). Investigation into the transcriptome analysis results for this gene from Iorizzo et al. [52] and Curaba et al. [53] confirms that *DcTTG1* is constitutively expressed, although a possible positive correlation between transcript abundance and total anthocyanin content was reported by Kodama et al. [95] in one cultivar. This suggests that *DcTTG1* may exist only as a functional gene in carrots, and could play a central role in the formation of the MBW complex similar to that observed in *Arabidopsis*. Although these results are preliminary, they provide directions to further study the MBW protein complex in carrots, and its impact on the regulation of the anthocyanin biosynthetic pathway.

The regulation of anthocyanin metabolism ends with their transport into the vacuole, a process which involves Glutathione S-transferases (GSTs) [102,103]. *DcGST1* (*DCAR_003401*), which co-localized with RTPE-Q2 QTL, was recently identified as being upregulated in two purple carrot cultivars [67], and its expression could be directly regulated by an A-MYB as it was found in *Arabidopsis* and apple plants [104,105]) (Table 3; Supplementary Table S4). In fact, based on the cultivar- and tissue-specific expression patterns, DcMYB113 likely controls the expression of *DcGST1*, and does this independently of DcMYB7 [71,72]. Interestingly, *DcMYB113* is also co-expressed with *DcMATE1* (*DCAR_031151*), another potential anthocyanin transporter [72,102]. A-MYBs are central to the regulation of anthocyanin biosynthesis genes, however the molecular mechanisms controlling their activity during carrot development remain largely unknown.

## 3. External Factors Affecting Anthocyanin Accumulation and Profile in Carrots and Other Plant Species

Phenolic compounds such as anthocyanins are essential to the interactions between plants and their environments [106]. Although some organs of some plant taxa can synthesize and accumulate anthocyanins in a nearly constitutive fashion (e.g., black carrots and red grapes), in other plants the accumulation of anthocyanins may reflect an adaptive response to adverse environmental conditions, and such accumulation of phenolics is considered an indicator of plant stress. Chemical elicitors or abiotic stresses could induce the accumulation of anthocyanin in carrots, and be an effective alternative and/or complement to breeding [107].

Various plant species show a similar anthocyanin induction in response to the same stresses, suggesting that the molecular mechanisms controlling anthocyanin stress responses are, at least partially, conserved among land plants. Temperature, light, nutrients and water intake are all environmental conditions that can affect anthocyanin biosynthesis [62,102,108]. Although not completely understood, their accumulation in response to abiotic stress has been associated with an increase in plant survival rate, which is likely due to their protective role against reactive oxygen species (ROS) [86,108,109]. Abiotic stress signaling was shown to affect the activity of several EBGs and LBGs genes in many plant species, coinciding with the production of anthocyanin. Such coordinated action likely involves the regulation of components of the MBW complex, and in particular A-MYBs and A-bHLHs [61,62,86,97,110,111].

Here we have summarized the effect of external factors on anthocyanin accumulation in carrots, and a few examples from some other species (Table 4). The treatment of carrot plants with exogenous phytohormones could also affect the production of anthocyanin. Indeed, foliar-applied ethephon, a precursor of ethylene (ET), enhances the content of anthocyanin and total phenolic compounds in Deep Purple carrot roots by about 25%, indicating that the production of anthocyanin can be increased even in black carrot varieties already containing a high level of anthocyanins [112]. In blueberries, apples and lettuce, the exogenous application of jasmonic acid (JA) increases the total phenolics content and antioxidant capacity [113,114,115]. In *Arabidopsis*, both abscisic acid (ABA) and JA promote the biosynthesis of anthocyanin in the presence of sucrose, while gibberellic acid (GA) and ET repress anthocyanin production [116,117,118,119]. The exogenous application of sucrose, which is perhaps the most potent inducer of anthocyanin biosynthesis, was reported in several plant species [120,121,122,123,124,125]. Although the effect of sucrose treatment in carrot has never been reported in planta, a significant increase of anthocyanin accumulation up to 7.5-fold was observed in carrot cell cultures [126,127] (Table 4). In *Arabidopsis*, the largest contributor of sucrose-induced anthocyanin accumulation was found to be *PAP1*, an ortholog of *DcMYB6* and *7* [56,128]. Interestingly, the transcript level of one of its homologs, *PAP2*, was found to increase about 1000-fold in response to nitrogen deficiency, and was proposed to be the mean mediator of anthocyanin accumulation in response to this stress [129], indicating the specification of A-MYBs to different stress-response pathways. A-MYBs’ activity can also be stress-regulated at the post-transcriptional level, as shown by the ubiquitination and degradation of MdMYB1 in response to increased nitrogen intake in apple tissue cultures [130]. Coherently, nitrogen concentration was shown to affect the production of anthocyanin in carrot cell cultures, but conflicting results were obtained from two independent studies, with both positive and negative effects of nitrogen being observed [126,127]. Altering the source of nitrogen by modifying the balance of ammonium to nitrate significantly affects the production of anthocyanin in carrot cell cultures, with a 1:4 ratio being optimum [127]. Additionally, reducing phosphorous intake was shown to enhance the production of anthocyanin in both carrot cell culture and *Arabidopsis* [109,126].

The application of postharvest abiotic stresses to carrots, such as wounding, could promote the accumulation of purple pigments. The activity of DcPAL1, along with the expression of *DcPAL1*, *DcC4H1* and *Dc4CL3-1*, significantly increases in wounded carrots, therefore promoting the metabolism of phenylpropanoids and the production of many of the phenolic defense compounds [133,134,135,140]. Wounding stress increases, by 75%, the total phenolic content in shredded carrots stored at 15 °C for 6 days [132]. Combining wounding with additional stresses such as heat, UV light, hyperoxia or the application of phytohormones and herbicide can synergistically increase the accumulation of phenolics in carrots [107,132,135,136,139,140,141]. For example, excess oxygen [107], higher storage temperatures [138], UV-B radiation [137] and ET [132] can enhance the content of total phenolics in wounded carrots 3.5-, 4.8-, 3.2- and 1.7-fold, respectively. Interestingly, ET and hyperoxia have little to no effect on non-wounded harvested carrots [132,135]. Although no detailed information exists concerning the anthocyanin profile in wounded carrots, the activation of the phenylpropanoid pathway and enhanced antioxidant capacity suggest that anthocyanins could be produced by wounding in some genetic backgrounds [132,134,135,137,138]. This correlates with the observation that combined light and mechanical stress can increase by, about 50%, the accumulation of anthocyanin in carrot cell cultures [142]. Additionally, the increases level of organic acids, such as ferulic acid, in response to wounding could improve anthocyanin stability through co-pigmentation effect [132,134,143].

Despite the observed effects of sugars, minerals and phytohormones on anthocyanin accumulation, to date no study has investigated the interaction between these external factors and anthocyanin-related genes in carrot. Extensive variations of anthocyanin-related TFs have been identified, and polymorphism within their promoter regions, such as was reported for *DcMYB7* alleles, suggests that a range of sensitivity to stress-induced anthocyanin responses could be observed between carrot cultivars [71,128]. Additionally, as observed in other plant species, exposure to various abiotic stress conditions could differentially affect the regulation of structural genes, and therefore the composition and tissue localization of anthocyanins being produced [108,123,144,145,146]. Two UDP-GT enzymes contribute to cold, salt and drought stress tolerance via modulating anthocyanin accumulation in *Arabidopsis* [131]. Such an osmotic stress response can be induce by mannitol, which promotes anthocyanin accumulation in both *Arabidopsis* and carrots [126,131]. Understanding the mechanism controlling anthocyanin’s response to stress would help us in developing new strategies to maximize the use of carrot as a natural colorant. Combined stresses can have an additive effect on the anthocyanin accumulation, and evidence from *Arabidopsis* suggests that the regulation of anthocyanin biosynthesis integrates independent and reversible stress-induced pathways. For example, in *Arabidopsis*, the accumulation of anthocyanin in response to salt stress is controlled independently of other stresses, such as high light, low phosphate limitation, high temperature or drought [147], and nitrogen and phosphorus depletion were found to trigger anthocyanin production through distinct pathways [109]. Most abiotic stress studies in carrots are postharvest, and more research to understand their effects on anthocyanin-regulatory genes during the development of the storage root is needed. For instance, extended growth was shown to significantly increase the concentration of anthocyanin in the storage root of the ‘Deep Purple’cultivar [148].

## 4. Perspectives

### 4.1. Advancing Molecular and Biotechnology Tools to Develop Carrot Cultivars That Maximize Anthocyanin Yield in Product Performance and Stability

Classical plant breeding approaches have succeeded in improving the productivity and quality of carrots for producers and consumers over the last century [149]. A significant focus in breeding has been placed on breeding for male sterility, disease resistance, vernalization requirement, root morphology and carotenoid content, with limited interest in anthocyanin-related phenotype/profile. Given the growing interest in multi-colored carrots and their nutritional profile, a few purple carrot cultivars have been released over the last few years [13,150]. However, as carrot breeding programs move forward, the expansion of carrot global markets and a broader range of consumer traits will require attention, including those related to anthocyanin accumulation for use as a natural food colorants. To improve anthocyanin content, profile and stability in well-established classic carrot breeding strategies can be adopted, and future work will need to focus on expanding molecular tools to facilitate the incorporation of multiple phenotypes into new cultivars. Commercial breeders today use molecular markers to implement Marker-Assisted Breeding (MAB) as a strategy for the effective improvement of multiple traits in plants [151]. MAB includes marker-assisted selection (MAS), marker-assisted recurrent selection (MARS), marker-assisted backcrossing (MABC), and genome-wide selection (GWS) or genomic selection (GS) [151]. Establishing DNA marker assays that can effectively be used for MAB is critical in order to bridge the gap between researchers discovering new QTLs and gene-trait associations, and breeders using this knowledge to make informed breeding decisions. Besides MAB, emerging biotechnology applications, such as gene editing, promise opportunities to effectively integrate desired traits into new cultivars. The application of this biotech method demands a more detailed understanding of the molecular mechanisms controlling desirable traits.

As described in this review, the number of marker trait association studies concerning anthocyanin in carrots has increased considerably over the last few years, leading to the identification of multiple genes controlling major QTLs and simply-inherited traits. Despite these advances, very few DNA marker assays targeting anthocyanin or other traits have been developed for carrots, thus limiting the implementation of MAB in carrot breeding programs. For example, a cleaved amplified polymorphic sequences (CAPS) marker has been developed for the *Y2* locus controlling carotenoid accumulation [152]. Similarly, a PCR-based marker, targeting the *DcSCPL1* gene controlling anthocyanin expression associated with the *Raa1* locus, is able to differentiate the low and high acylation alleles [53]. However, all of these markers were tested in just a few mapping populations, representing a very narrow genetic diversity. Therefore, it is unknown if these DNA assays would be successful in other genetic backgrounds. The reliability of molecular markers in predicting the target trait depends on their close linkage with the mutation that effects the phenotype [153]. This is particularly important in outcrossing species, such as carrots, in which Linkage Disequilibrium (LD) decays rapidly [152], and identifying causal mutations that are present in perfect LD with the phenotype is critical for effective MAB application. Considering the high genetic variation existing in the carrot germplasm [152], it is common that PCR primers developed for one mapping population do not work in other genetic backgrounds. In order to implement MAB, these QTLs need to be validated in wider breeding populations and germplasm collections, and under different growing conditions. However, all of these PCR-based marker methods are low throughput, relatively expensive, and are labor intensive, which limits their use in carrot breeding programs. Multiple cost-effective low-density genotyping assays, like TaqMan, KASPar and semi-thermal asymmetric reverse PCR (STARP) [58], are currently available, and could be used to develop a panel of allele-specific assays from functional genes or QTLs. This panel could enable MAB in a high-throughput, cost-effective fashion in carrot. To implement MAB for the improvement of carrot anthocyanins, future work should focus on identifying the causal mutations underlying anthocyanin-related QTLs, then should design and validate high-throughput DNA assays to be applied to carrot breeding materials.

As summarized in this review, the genes controlling four major anthocyanin QTLs (*P*_1_, *P_3_*, RTPQ-2 and *Raa1*) have been identified and functionally characterized. However, candidate genes for >37% of the anthocyanin QTLs detected in carrots to date have not yet been identified. The integration of QTL studies and the genomic data presented in this review has helped identify candidate genes for some minor QTLs, and establish a foundation for future studies to characterize these genes. Moreover, a Genome Wide Association Study for anthocyanin expression in the different root tissue layers is needed in order to better understand the genetic and molecular mechanism controlling this trait, and identify the ideal allele combination that can maximize anthocyanin content in the carrot root. Besides allelic genes controlling anthocyanin QTLs, it will also be important to better understand the overall molecular mechanisms involved in controlling anthocyanin biosynthesis, storage and degradation in carrots during normal development, as well as in response to stressors that are not under allelic control. The differential regulation of structural enzymes under specific stresses, such as the branch point or end of pathway enzymes involved in anthocyanin decoration, could easily affect the profiles of polyphenolics being produced. Expanding this knowledge will open the opportunity to develop biotech-based solutions, such as gene editing or transformation, in order to accelerate the development of cultivars that can maximize anthocyanin colorant yield and stability. As summarized in this review, a number of studies of carrots used transgenic and gene editing approaches to characterize the function of genes controlling anthocyanin biosynthesis [71,72,83,98]. Genetic engineering can offer new possibilities for the control of gene expression, not only to increase anthocyanin production, but also to reduce their degradation and maximize their stability [62]. Because of their global impact on structural gene regulation, *A-MYB* and *A-bHLH* genes represent targets of choice that have been successfully used for engineering anthocyanin accumulation, by either overexpressing or knocking down their expression [86,92,110,154,155]. However, the targeting of specific structural genes could be used to favor the production of certain types of anthocyanins [63,81,156,157,158]. Chimeric RNA interference (RNAi) technology is on the rise [159], and has been used to modulate anthocyanin biosynthesis in the fruit, flowers and roots of other plant species, sometimes by targeting multiple biosynthetic genes at once [79,80,104,155,156,157,160]. So far in carrots, RNAi has been successfully used to silence the pathogenesis-related protein-coding genes, so as to reduce allergenicity [161], and two carotene desaturase genes, in order to characterized their function [162], highlighting the potential of this technique that could be used in carrots to selectively suppress the activity of multiple biosynthesis enzymes, and redirect metabolic flux toward the production of polyphenolic compounds of agricultural interest. Future efforts should focus on expanding the applications of these techniques, so as to modulate the expression of genes that can maximize the yield and stability of the anthocyanin extracts.

In comparison to a transgenic approach, a gene editing technique such as CRISPR-Cas9 offers the advantage that gene-edited crops are not considered genetically modified organism (GMO) in some countries, such as the US, where the demand for natural food colorants such as anthocyanins is high. Indeed, the use of GMO crops as a source of natural pigments may be inconsistent with consumer interests. However, carrot cultivars engineered with either the transgenic or gene editing approach have not been reported so far, but their development is possible.

Genetic engineering can also be used to develop desired cell lines for in vitro systems. Bioreactor technologies have been well developed for carrot cell culture, and anthocyanin production in cultured cells has been investigated [163,164]. Bioreactors may provide a unique approach to pigment production from plant cells, which bypasses field-grown plant materials. Theoretically, the production of high-anthocyanin cell suspensions or embryonic tissues in bioreactors is possible, but no reports have indicated that such production methods are of economic significance.

### 4.2. Exploring the Diversity of Co-Pigmentation to Enhance Anthocyanin Product Performance and Stability

For the food colorant industry, the ability to retain or enhance natural pigment properties is a major priority in product application. Similarly, the reduction of usage rates remains critical, as relative to synthetic colors, anthocyanin-based color systems often have a weaker color intensity, requiring higher dosages to attain similar shades [165]. Of the potential approaches, co-pigmentation is a method that is observed to intensify the anthocyanin color by shifting the maximum absorption wavelength in the visible range to a higher wavelength, and it also increases extinction efficiency [166]. Co-pigmentation naturally occurs with some anthocyanin compounds and in select food matrices, such as juice and wine [143,167,168]. This interaction between anthocyanins, and between anthocyanins and other phenolics, has been reported to have a stabilizing effect, either through intermolecular or intramolecular interactions, by the accumulation and assembling of the hydrophobic acyl moiety covalently bound to sugar and a flavylium nucleus [169,170]. In the co-pigmentation of anthocyanins by colorless phenolics, a van der Waals interaction protects the C-2 of the flavylium chromosphore from nucleophilic attack by water, which prevents color loss but may also alter its hues and attributes depending on the co-pigments and the environment available for interaction [171]. The co-pigmentation of purple sweet potato anthocyanins with chlorogenic acid and other plant phenolics increased the p*K*_H_ estimate values of anthocyanins from 3.28 to 4.71, extended the pH range from 2.6 to 4.6, and increased the variation in color hues [172]. Due to the associated health benefits and co-pigmentation properties of chlorogenic acid, improving the chlorogenic acid content in eggplant is a current breeding goal [173,174]. In carrots, it has been hypothesized that the amounts and types of polyphenolics in black carrots may enable co-pigmentation in selected food products [175]. For instance, the use of an external source of chlorogenic acid to co-pigment a black carrot anthocyanin solution improves pigment stability [176]. In black-colored dahlia plants, FNSI has been shown to play a key role in regulating the flux of flavones and enhancing purple color intensity by co-pigmentation with anthocyanin [78]. A similar mechanism could exist in carrots, which accumulate flavones and in which FNS1 plays a critical role in balancing the metabolic flux of phenylpropanoids. This could contribute to enhancing color stability through co-pigmentation. Furthermore, multiple studies of carrots have indicated that carrot roots contain extensive variations of phenolic acids, largely represented by chlorogenic, *p*-coumaric, caffeic and ferulic acids [28,47,177], with purple carrots containing up to 16 times more polyphenolics than other colored carrot roots [31,177]. Despite the potential use of phenolics as co-pigmentation agents to enhance color intensity or prevent color loss, to date no study in carrot has investigated the potential to select cultivars with high phenolics and high acylated anthocyanins simultaneously. Further, the inability to derive an understanding of the chemistries involved in these compounds through typical water-based extractions, and their functioning in product applications, limits our ability to employ this potentially game changing interaction. Future work should focus on exploring the genetic mechanisms controlling phenolic content, while more studies are needed in order to investigate the role of phenolics in the co-pigmentation of black carrot anthocyanin extracts, and their role in food products for their ability to enhance color intensity and stability.

## Figures and Tables

**Table 1:** Anthocyanin detected in carrots to date with approximate HPLC retention times and molecular masses.

Compound	Abbreviation	RT	MW
Cyanidin 3-xylosylglucosylgalactoside	Cy3XGG	14.0	743
Cyanidin 3-xylosylgalactoside	Cy3XG	15.1	581
Cyanidin 3-xylosyl(sinapoylglucosyl)galactoside	Cy3XSGG	15.4	949
Cyanidin 3-xylosyl(feruloylglucosyl)galactoside	Cy3XFGG	16.0	919
Cyanidin 3-xylosyl(coumuroylglucosyl)galactoside	Cy3XCGG	16.4	889
Pelargonidin 3-xylosyl(feruloylglucosyl)galactoside	-	21.8	903
Peonidin 3-xylosylgalactoside	-	22.3	595
Peonidin 3-xylosyl(sinapoylglucosyl)galactoside	-	22.7	963
Peonidin 3-xylosyl(feruloylglucosyl)galactoside	-	23.3	933

RT is retention time (min) of cyanidin glycosides, as reported for the chromatographic procedure described by Kurilich, et al. [21]; whereas for pelargonidin and peonidin glycosides, RT are as reported by Montilla, et al. [6]. MW is molecular weight.

**Table 2:** Summary of studies investigating the level of degradation/stability of anthocyanin obtained from black carrot.

Sample	Storage Parameters	Key Findings	Citation
Fermented black carrot beverage (Shalgam)	T = 4, 25, and 40 °C ST = 90 days	Anthocyanin degradation rate was significantly higher at 40 °C storage temperature; Anthocyanin degradation rate at 4 °C and 25 °C was not significantly different AA were significantly more stable than NAA	[42]
Black carrot concentrate	T = 4, 20 and 37 °C ST = based on t1/2 Brix = 30, 45 and 64 pH = 2.5, 3, 4, 5, 6, 7	Degradation of anthocyanin stored at 37 °C was much faster compared to storage at 4 °C (t1/2 = 4.0–4.5 and 71.8–215 weeks, respectively) Significant decrease in anthocyanin stability was observed at pH values above 5.0	[44]
Black carrot juice concentrate	T_1_ = −23, 5 and 20 °C ST_1_ = 319 days T_2_ = 30 °C ST_2_ = 53 days	AA exhibited higher stability during storage; under sub-freezing conditions, anthocyanin degradation was minimal	[46]
Turkish delight colored with black carrot juice concentrate	T = 12, 20 and 30 °C ST = 5 months	At higher temperatures anthocyanin degradation rate was higher	[45]
Soft drink colored with black carrot extract	T = 4, 20, 30 and 50 °C ST = 60 days	At 4 °C no degradation was detected Anthocyanin from black carrot degraded more slowly than blackberry and aҫai anthocyanin	[48]
Sliced purple carrots	T = 2, 4 °C ST = 4 weeks	No significant difference in anthocyanin content were detected between 2 and 4 °C storage temperatures	[49]
Black carrot jams and marmalades	T = 4 and 25 °C ST = 20 weeks	The reduction of anthocyanin content in samples stored at 4 °C was lower than that of samples stored at 25 °C AA were significantly more stable than NAA	[47,50]
Black carrot concentrate	pH = 3, 4, 5	Cy3SGG was found to exhibit a lower visual detection threshold and a higher pH than Cy3FGG and Cy3XCG	[6]
Black carrot concentrate	pH = 1, 2, 3, 4, 5, 6, 7, 8, 9, 10 ST = 30 min	Degradation rates of anthocyanin increased with pH	[8]

ST = Storage time; T = Temperature; ST_1_ and ST_2_ Storage time used for experiment 1 and 2 respectively; T_1_ and T_2_ = Temperatures used for experiment 1 and 2 respectively; t1/2 time needed for 50% degradation of anthocyanin.

**Table 3:** Subset of carrot anthocyanin structural and regulatory genes. Functionally characterized genes are in bold. The Overlapping Anthocyanin QTLs column shows every QTL or genomic region containing the gene by indicating the type of trait with the corresponding number of associated QTLs, and the number of genotypes analyzed is in brackets. The Expression in Purple Tissue column shows the type of transcriptional regulation, Up (UP), Down (DW) or not differentially expressed (X), occurring in purple versus non-purple root (R) or petiole (P) tissue, as well as the purple genotype(s) in which it was observed: B9547, B7262 [55]; 5394, 95710, 5723 [52,53]; 3242 [34]; Deep Purple (DPP), Purple 68 (PP68), Purple Haze (PPHZ), Tianzi2hao (TZ2H), Zibacun Solid purple (ZBC-S), Zibacun Peridermal purple (ZBC-P), Cosmic Purple (CPP), transgenic overexpressing *DcMYB7* (35SMyb7), transgenic overexpressing *DcMYB113* (35SMyb113) [67,71,72,82].

Function	Name	Locus ID	Overlapping Anthocyanin QTLsn	Expression in Purple Tissue	
				Type	Genotype
**Regulatory Genes**	*DcMYB5*	DCAR_024737	XSGG(1,1); Ph-SumNAA(1,1)	DW(R)	PP68
	*DcMYB6*	DCAR_000385	P_2_(1,1); P_3_(1,2); RPTE(1,1); TotalANT(1,1); SumAA(2,1); SumNAA(1,1); G(1,1); GG(2,1); SGG(3,2); FGG(3,2); CGG(2,2); Ph-TotalANT(2,1); Ph-SumAA(2,1); Ph-SumNAA(3,1); Ph-G(2,1); Ph-GG(3,1); Ph-CGG(3,1); Ph-SGG(2,1); Ph-FGG(1,1)Xy-TotalANT(1,1); Xy-SumAA(1,1); Xy-SumNAA(1,1); Xy-G(1,1); Xy-CGG(1,1); Xy-SGG(1,1); Xy-FGG(1,1); PAP(1,2); Phloem(1,1); XAP(1,1)	UP(R)	5394, 3242, DPP, PP68, TZ2H, ZBC-S, ZBC-P, CPP, 35SMyb7
				DW(P)	5723, 95710
	*DcMYB7*	DCAR_010745		UP(R)	7280, 5394, 95710, 3242, DPP, PP68, TZ2H, ZBC-S, ZBC-P, CPP, 35SMyb7
				UP(P)	5723, 95710, 35SMyb7
	*DcMYB10*	DCAR_010749		UP(P)	5723
	*DcMYB11*	DCAR_010751		UP(P)	5723, 95710
	*DcMYB113*	DCAR_008994	P_1_(1,1); Ph-TotalANT(2,1);Ph-SumAA(2,1); Ph-SumNAA(2,1); Ph-G(2,1); Ph-GG(2,1); Ph-CGG(3,1); Ph-SGG(2,1); Xy-CGG(1,1)	UP(R)	PPHZ, 35SMyb113
	*DcMYB17*	DCAR_007287	Ph-GG(1,1)	UP(R)	PP68
	*DcMYB19*	DCAR_015602	Ph-FGG(2,1)	UP(R)	PP68
	*DcMYB22*	DCAR_018882	Ph-TotalANT(1,1)	DW(R)	PP68
	*DcMYB1R1-1*	DCAR_026095	-	DW(R)	PP68
	*DcMYB1R1-2*	DCAR_024503	SGG(1,1); Ph-SumNAA(1,1)	DW(R)	PP68
	*DcbHLH3*	DCAR_002739	RTPE(1,1)	UP(R)	5394, 95710, DPP, PP68, PPHZ, TZ2H, ZBC-S, ZBC-P, CPP, 35SMyb7, 35SMyb113
	*DcTTG1*	DCAR_020377	-	X(R)	5394, 95710
				X(P)	5723, 95710
	*DcGST1*	DCAR_003401	RTPE(1,1)	UP(R)	DPP, PP68, PPHZ, CPP, 35SMyb113
**Structural Genes**	*DcPAL4*	DCAR_017697	-	UP(R)	5394, 95710, DPP, PP68, TZ2H
	*DcC4H1*	DCAR_018641	-	UP(R)	5394, 95710, DPP, PP68, TZ2H
	*Dc4CL3-1*	DCAR_021385	-	UP(R)	95710, DPP, PP68, TZ2H
				DW(P)	95710
	*Dc4CL3-2*	DCAR_025617	-	UP(R)	95710
	*DcCHS1*	DCAR_030786	-	UP(R)	B9547, B7262, 5394, 95710, 3242, DPP, PP68, PPHZ, TZ2H, ZBC-S, ZBC-P, CPP, 35SMyb7, 35SMyb113
				UP(P)	5723
	*DcCHI1*	DCAR_027694	-	UP(R)	5394, 95710, 3242, DPP, PP68, PPHZ, TZ2H, ZBC-S, ZBC-P, CPP, 35SMyb7, 35SMyb113
				UP(P)	5723
	*DcF3H1*	DCAR_009483	-	UP(R)	B9547, B7262, 95710, 3242, DPP, PP68, PPHZ, TZ2H, ZBC-S, ZBC-P, CPP, 35SMyb7, 35SMyb113
				UP(P)	5723
	*DcF3’H1*	DCAR_014032	Ph-TotalANT(2,1); Ph-SumNAA(3,1); Ph-G(3,1); Ph-GG(4,1)	UP(R)	95710, 3242, DPP, PP68, PPHZ, TZ2H, ZBC-S, ZBC-P, CPP, 35SMyb7, 35SMyb113
	*DcDFR1*	DCAR_021485	-	UP(R)	B9547, B7262, 5394, 95710, 3242, DPP, PP68, PPHZ, TZ2H, ZBC-S, ZBC-P, CPP, 35SMyb7, 35SMyb113
				UP(P)	5723, 9571
	*DcUSAGT*	DCAR_029082	-	UP(R)	7280, 5394, 95710
	*DcLDOX1*	DCAR_006772	-	UP(P)	DPP, PP68, PPHZ, TZ2H, ZBC-S, ZBC-P, CPP, 35SMyb7, 35SMyb113 5723, 95710
	*DcUCGXT1*	DCAR_021269	SGG(1,1)	UP(R)	7280, 5394, 95710, DPP, PP68, PPHZ, TZ2H, ZBC-S, ZBC-P, CPP, 35SMyb7, 35SMyb113
	*DcUCGalT1*	DCAR_009912	TotalANT(1,1); SumAA(1,1); XFGG(1,1); Ph-CGG(2,1)	UP(R)	3242, 7280, 5394, 95710, DPP, PP68, PPHZ, TZ2H, ZBC-S, ZBC-P, CPP, 35SMyb7, 35SMyb113
	*DcSCPL1*	LOC108214129	Raa1(1,1); XGG-(1,1); SGG(1,1); FGG(1,1); Ph-CGG(2,1)	UP(R)	7280, 5394, 95710, DPP, PP68, PPHZ, TZ2H, ZBC-S, ZBC-P, CPP, 35SMyb7, 35SMyb113
	*DcSCPL12*	LOC108227197	Ph-SGG(2,1)	UP(R)	95710
	*DcSCPL13*	LOC108227196		UP(R)	5394, 95710
	*DcSCPL14*	LOC108227198		UP(R)	7280
	*DcSCPL15*	LOC108192824		X(R)	5394, 95710
	*DcBAHD39*	LOC108196041	SumAA(1,1); SumNAA(1,1); G(1,1); GG(1,1); SGG(1,1); FGG(1,1); CGG(1,1); Ph-TotalANT(2,1); Ph-SumNAA(1,1); Ph-G(1,1); Ph-GG(%)(2,1); Ph-SGG(1,1); Ph-FGG(1,1)	UP(R)	95710

**Table 4:** Examples of external factors that can enhance the biosynthesis of anthocyanins and other phenolics in carrots, and their effects on other plant species.

External Factor	Phenolic Quantified **	Species	Tissue	Reference
Ethephon	TA^65%^; TP^25%^	Black Carrot	root	[112]
Sucrose	TA^756%^	Carrot	Callus	[126]
	TA^225%^	Carrot	Callus	[127]
	TA^600%^	*Arabidopsis*	Seedling	[117]
	TA^>600%^	*Arabidopsis*	Seedling	[124]
	TA^570%;^ A5^>2000%;^ A8^>600%;^ A9^>4000%^; A11^>300%^	*Arabidopsis*	Seedling	[123]
	TA^300%^	Grape	Cell culture	[120]
	TA^1500%^	Radish	Hypocotyl	[121]
	TA^>60%^	Petunia	Seedling	[125]
Mannitol^+SUC^	TA^156%^	Carrot	Callus	[126]
Mannitol	TA^60%^	*Arabidopsis*	Seedling	[131]
N limitation	TA^160%^	Carrot	Callus	[126]
	TA^4400%^	*Arabidopsis*	Seedling	[129]
	TA^750%;^ quercetin^700%;^ kaempfero^l200%;^ cyanidin^>3000%^	*Arabidopsis*	Seedling	[109]
Pi limitation	TA^120%^	Carrot	Callus	[126]
	TA^500%^	*Arabidopsis*	Seedling	[109]
Wounding	TP^750%^; CHA^500%^; FA^165%^; IC^290%^	Carrot	Root *	[132]
	TP^800%^; CHA^500%^; IC^1300%^	Carrot	Root *	[133]
	TP^252%^; CHA^1000%^; 3,5-diCQA^80%^; FA^>1000%^; IC^>1000%^	Carrot	Root *	[134]
	TP^287%^; 3-CQA^700%^; 3,5-diCQA^>3500%^; 4,5-diCQA^150%^; FA^140%^	Carrot	Root *	[135]
ET^+W^	TP^65%^; CHA^90%^; IC^1860%^	Carrot	Root *	[132]
UV^+W^	TP^143%;^ CHA^600%;^ FA^100%;^ IC^60%^	Carrot	Root *	[136]
	TP250%; CHA750%	Carrot	Root *	[137]
Hyperoxia^+W^	TP^30%^; 3-CQA^75%^; 3,5-diCQA^75%^; 4,5-diCQA^100%^; FA^70%^	Carrot	Root *	[135]
High Temp^+W^	TP^150%^	Carrot	Root *	[138]
Glyphosate^+W^	SA^938%^; CHA^1988%^; FA^938%^	Carrot	Root *	[139]

+SUC External factor tested in sucrose-enriched conditions. +W External factor tested in addition to wounding. * Post harvest study. ** Subset of phenolic compounds quantified in the corresponding study, which showing a significant increase in response to the elicitor treatment. The percentage of maximum increase (in some case estimated from data chart) caused by the elicitor is indicated in superscript; the sign “>” indicates that the compound was not detectable in the control sample. Total phenolics (TP), total anthocyanin (TA), total flavonoid (TF), shikimic acid (SA), chlorogenic acid (CHA), ferulic acid (FA), isocoumarin (IC), 4,5-dicaffeoylquinic acid (4,5-diCQA), 3-O-caffeoylquinic acid (3-CQA), 3,5-dicaffeoylquinic acid (3,5-diCQA); *Arabidopsis* cyaniding-based anthocyanin type A5 (A5), A8 (A8), A9 (A9), A11 (A11).

## Supplementary Materials

The following are available online at https://www.mdpi.com/2073-4425/11/8/906/s1, Figure S1: Phylogenetic analysis of candidate carrot TTG1. Phylogenetic relationships of selected WD40 proteins. Phylogenetic tree was constructed using the neighbor-joining method by the MEGA6 software. The reliability of the trees was tested using a bootstrapping method with 1000 replicates. Numbers indicated bootstrap values for 1000 replicates. Four carrot proteins (DCAR_020377, DCAR_000204, DCAR_014316 and DCAR_016686) were included in this analysis. DCAR_020377 is orthologous and syntenic (Iorizzo et al., 2016) to *Arabidopsis* thaliana AtTTG1, while DCAR_000204, DCAR_014316 and DCAR_016686 are the most similar to DCAR_020377 (not orthologous or syntenic). The GenBank accession numbers of the WD40 protein sequences were listed as follows: *Prunus* persica PpTTG1 (ACQ65867), *Malus* domestica MdTTG1 (GU173813), *Prunus* persica PpTTG1 (ACQ65867), *Punica* granatum PgWD40 (HQ199314), *Vitis* vinifera VvTTG1 (NP_001268101), *Fragaria* x ananassa FaTTG1 (AFL02466), *Gossypium* hirsutum GhTTG1 (AAM95641), GhTTG3 (AAM95645), *Petunia* hybrida PhAN 11 (AAC18914), *Arabidopsis* thaliana AtTTG1 (Q9XGN1), *Arabidopsis* thaliana AtLWD1 (Q9LPV9) and *Arabidopsis* thaliana AtLWD2 (Q38960). AtLWD1 and AtLWD2 were used as outgroups. Table S1: Description of QTL and simply-inherited traits conditioning the presence and concentration of carrot anthocyanins. Table S2: QTL and simply-inherited traits conditioning the presence and concentration of carrot anthocyanins mapped in chromosome 3. Table S3: Mapped QTL conditioning the concentration of carrot anthocyanins, by chromosomes 1, 2, 4–9. Table S4: List of known carrot anthocyanin structural and regulatory genes. Column [F]: gene symbol annotation is based on the literature and blast results; SCPL and BAHD acyltransferases were numbered according to their location on the genome, from chromosome 1 to 9. Gene coordinates correspond to the locations of the gene models on the carrot v2 genome. Column [I] indicates if a gene clustered (“Yes”) or not (“No”) with known anthocyanin-related gene(s) from other species based on orthologous (“Orth”), phylogenetic (“Phyl”) and/or weighted gene co-expression network (WGCN) analysis. Column [K] indicates when transcripts were detected in at least one tissue sample (root or petiole); “X” = gene expressed; “0” = gene non expressed or below 1 RPKM; “-” = no data available. Columns [L–V]: deregulation associated with purple pigmentation derived from 9 studies; the number of purple genotypes and the type of method used is written in brackets for each study; labeling indicate when a gene was found to be Up (“UP”) or Down (“DW”) regulated in at least one comparable purple vs. non-purple tissue. Columns [W–Z] indicate the genome duplication associated to each gene; *Genome Duplication Modes (GDM): “T” = tandem duplication; “P” = Proximal duplication; “D” = Dispersal duplication; “W” = Whole genome duplication. ** Number in each column indicate the number of genes detected in each of the carrot duplicated blocks, with paralogous gene ID indicated in parenthesis.
