# Supplementary material for: Carrot Anthocyanins Genetics and Genomics: Status and Perspectives to Improve Its Application for the Food Colorant Industry
Source: Genes (Basel). 2020 Aug 7;11(8):906. doi: 10.3390/genes11080906 (PMC7465225; doi:10.3390/genes11080906)
Supplement: Supplementary file 1 [file genes-11-00906-s001.zip › Supplementary Figure 1.docx]

**Supplementary Figure 1.**
